# Defining the methodological challenges and opportunities for an effective science of sociotechnical systems and safety

**DOI:** 10.1080/00140139.2015.1015622

**Published:** 2015-04-02

**Authors:** Patrick Waterson, Michelle M. Robertson, Nancy J. Cooke, Laura Militello, Emilie Roth, Neville A. Stanton

**Affiliations:** ^a^ Human Factors and Complex Systems Group, Loughborough Design School, Loughborough University, LoughboroughLE11 3TU, UK; ^b^ Liberty Mutual Research Institute for Safety, Hopkinton, MA01748, USA; ^c^ College of Technology and Innovation, Arizona State University, USA; ^d^ Applied Decision Science, LLC, Cincinnati, OH45112, USA; ^e^ Roth Cognitive Engineering, Menlo Park, CA94025, USA; ^f^ Engineering and the Environment, University of Southampton, Highfield, SouthamptonSO17 1BJ, UK

**Keywords:** sociotechnical systems, human factors and ergonomics methods, macroergonomics, workplace design and evaluation

## Abstract

An important part of the application of sociotechnical systems theory (STS) is the development of methods, tools and techniques to assess human factors and ergonomics workplace requirements. We focus in this paper on describing and evaluating current STS methods for workplace safety, as well as outlining a set of six case studies covering the application of these methods to a range of safety contexts. We also describe an evaluation of the methods in terms of ratings of their ability to address a set of theoretical and practical questions (e.g. the degree to which methods capture static/dynamic aspects of tasks and interactions between system levels). The outcomes from the evaluation highlight a set of gaps relating to the coverage and applicability of current methods for STS and safety (e.g. coverage of external influences on system functioning; method usability). The final sections of the paper describe a set of future challenges, as well as some practical suggestions for tackling these.

**Practitioner Summary:** We provide an up-to-date review of STS methods, a set of case studies illustrating their use and an evaluation of their strengths and weaknesses. The paper concludes with a ‘roadmap’ for future work.

## 1. Introduction

The term ‘Sociotechnical Systems’ (STS) dates back to work carried out in the 1950s by a group of researchers at the London Tavistock Institute of Human Relations. The work of Eric Trist and Fred Emery initially focused on understanding the role of human skill and methods of working (e.g. team working) on productivity within coal mines (Trist and Bamforth 1951). One of the primary motivations for STS was to underscore the role of choice and organisational design in the interaction between people (the social system) and tools, technologies and techniques (the technical system – Weisbord 2012; Eason 2014). A core value of the STS approach is that, given the right choices, social and technical systems could be harmonised and balanced such that productivity, worker satisfaction and safety could be optimised in parallel (Cherns 1976, 1987; Clegg 2000). In more recent years, STS has influenced the development of a number of other different domains within human factors and ergonomics. In particular, STS forms the basis of macroergonomics and systems ergonomics. Macroergonomics is defined by Hendrick and Kleiner (2001) as ‘a top-down sociotechnical system approach to the design of work systems and the application of the overall work-system design of the human-job, human-machine, and human-software interfaces’. Systems ergonomics emerged within the UK and Europe in the 1960s and builds on STS in viewing complex systems, for example organisations, teams and types of technology, as composed of interrelated components, the properties of which are changed if the system is dissembled in any way (Katz and Kahn 1966).

During the 1960s and 1970s, work within STS focused on issues such as the impact of new technology on work organisation and jobs (e.g. Davis 1971) and its impact on the quality of working life (e.g. Davis and Trist 1974; Taylor 1977). Only later did the original STS influence on management systems and organisational dynamics come to be applied to safety management. Since that time, there has been a growing interest in applying concepts and constructs from STS to the broad domain of safety, including aspects of occupational risk, injury and health (e.g. Kleiner 2004; Carayon 2006) as well as work which attempts to understand the causes of large-scale accidents and disasters (Rasmussen 1997; Leveson 2012).

### 1.1 STS and safety: from technology to human factors methods

Hale and Hovden (1998), in their analysis of the historical development of the scientific study of safety, outline three separate ‘ages’. The first age covers the period from the nineteenth century up until the Second World War and involved the use of exclusively technical measures to prevent the occurrence of explosions and collapse of structures (e.g. technologies such as safety valves and machine guards). The second age (the ‘age of human factors’) was characterised by the integration of human factors with established methods for risk and safety analysis (e.g. probabilistic risk analysis). During the 1980s, interest in the role played by human factors increased and is reflected in the development of methods such as human reliability analysis (HRA – e.g. Swain and Guttman 1983; Kirwan 1994), Hazard and operability study (Kletz 1983) and failure modes and affect analysis (FMEA – Kirwan and Ainsworth 1992).

The late 1970s and early 1980s were also periods during which there was an increased focus on understanding the role of cognition in human decision-making, particularly in complex, high-risk domains such as nuclear power plants, aviation and military command and control (Hollnagel and Woods 1983; Klein, Calderwood, and Clinton-Cirocco 1986; Rasmussen 1986; Woods and Hollnagel 1987; Woods and Roth 1988a). This research thread provided the foundation for the field of Cognitive Systems Engineering. Cognitive Systems Engineering places an emphasis on studying ‘naturalistic decision-making’ in real-world contexts, requiring consideration of the broader sociotechnical context (Klein et al. 1993; Woods and Roth 1988b). It also led to the development of a number of methods for capturing and representing the cognitive and collaborative demands associated with the sociotechnical system as well as the knowledge and strategies that enable people to operate effectively as part of those systems. This includes the development of cognitive work analysis (CWA) methods (Rasmussen 1986; Vicente 1999; Read et al. 2014) and cognitive task analysis (CTA) methods (e.g. Klein, Calderwood, and MacGregor 1989; Roth and Woods 1988). See Roth (2008) for a discussion of the roots of CTA and CWA methods, and Bisantz and Roth (2008) and Roth and Bisantz (2013) for comprehensive overviews of CTA methods and CWA methods and applications.

**Figure 1:**
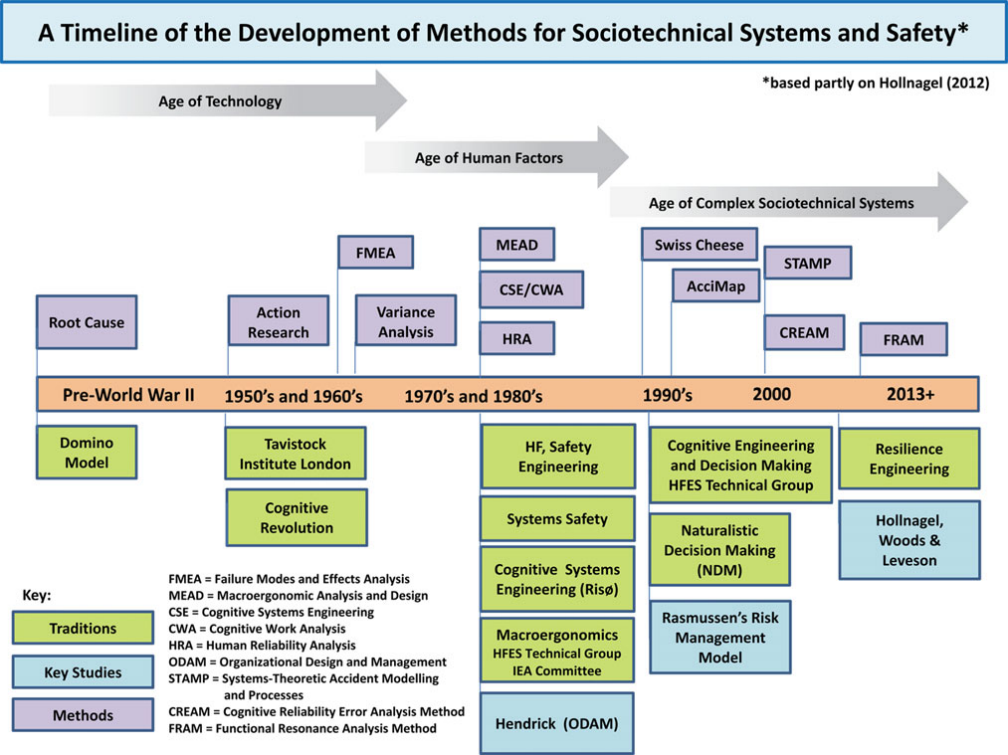
A timeline of the development of methods for sociotechnical systems and safety.

Since the 1990s, and against a background of accidents such as Chernobyl (1986), Zeebrugge (1987) and Challenger (1986), there has been an increasing focus on understanding the underlying causes of the failure of large-scale, complex STS. Hale and Hovden (1998) characterise this ‘third age’ of safety as shifting away from an exclusive focus on individual error and towards efforts to gain a better understanding of the management of safety, particularly in terms of what became commonly known as safety ‘culture’ and ‘climate’. A key part of this was the ‘rediscovery’ of the importance of STS theory and its relevance for safety (e.g. Robinson's 1982 application of STS principles to the design of safe systems). A second development was the recognition that the pace of technological developments along with increasing pressures on companies operating in commercially aggressive and competitive environments had raised the potential for major accidents and errors to occur (Rasmussen 1997; Kirwan 2001). Many of these developments are reflected in the development of methods which draw on STS theory and are linked to a variety of research traditions (e.g. Cognitive Systems Engineering; Naturalistic Decision Making – Figure 1).

### 1.2 The changing nature of safety and the need for new methods

Many of the other papers in this special issue (e.g. Carayon et al., Kleiner et al.) in common with other authors (e.g. Rasmussen 2000; Hollnagel 2008; Leveson 2012), describe a number of future requirements for STS and safety. These include the fact that twenty-first century safety requires us to understand the increased complexity and interconnectivity between systems and their elements. Rasmussen's (1997) work on a risk management framework for safety emphasised the rapid pace in which technology has changed and developed. In the decade since that paper, technology has become even more complex, and attempts to keep up with new developments have become an imperative, rather than simply something to be desired, in terms of the theory and practice of safety in organisations.

A similar challenge for the future will be dealing with the increasing complexity and intractability of STS. How can proactive safety or ‘resilience’ be achieved (Hollnagel, Woods, and Leveson 2006)? How can we evaluate the likely trade-offs that occur when organisations need to consider overall safety in terms of other issues such as system reliability, production costs, security and productivity (Wilson et al. 2009)? In the present environment, where governments and organisations face huge pressure to reduce costs and curtail budgets, these concerns are likely to take on more importance. All of these considerations will have implications for the development of methods for STS and safety. As one leading researcher put it:

The main problem in industrial safety today is that the majority of safety management and risk assessment methods are from 20 to 40 years old … [and] … may have been adequate for the systems that existed at the time they were developed, but are inadequate for present day systems. (Erik Hollnagel quoted in Wilson et al. 2009)

In this paper, we attempt to tackle some of these issues by examining current sociotechnical methods and assessing their suitability, both from a theoretical and practical standpoint, in dealing with present day safety issues, as well as future requirements. We address the following questions:

1. What types of methods already exist for studying and designing new interventions involving STS and safety? How can these be related to the various sub-disciplines and research traditions which make up human factors engineering (HFE)?
2. How well do current HFE methods fit the safety requirements of present-day and future STS?
3. What steps need to be taken in order to address the limitations of current HFE methods in dealing with the safety of STS in the future?

In order to provide answers to these questions, the paper has the following structure: Section 2 outlines a set of research methods which are linked to some of the research traditions described in Figure 1. One of the chief aims is to provide an overview (a ‘conceptual map’) of some of the main methods associated with research traditions rather than an exhaustive or comprehensive set of methods. Section 3 of the paper describes a set of six case studies which are used to illustrate some of these methods and provide a context for their application. In Section 4, an evaluation carried out by the authors of a selection of the methods is described. The remaining section of the paper (Section 5) uses the outcomes from constructing Table 1, the results from the evaluation, alongside our own reflections on work in this area, to identify a number of gaps in terms of coverage and practical utility. The paper concludes with a series of challenges and unresolved issues which could form the basis of future work with STS and HFE, alongside some practical suggestions for how this could be addressed.

**Table 1:** Traditions and conceptual underpinning theories, methods, data collection techniques, references, applications and relevant case studies.

			Data collection techniques								
Traditions, conceptual underpinning and theory	Framework and approach	Methods							Key references	Applications (references)	Case studies
STS theory, quality management and ergonomics	Participatory design variants: cooperative design	**Action research** Action research typically involves: (a) systematically collecting research data about an on-going system; (b) feeding these data back to the system and conducting a collaborative diagnosis of the data; (c) taking action based on the diagnosis, and (d) evaluating the results of the action	X	X				X Work-shops	Ramirez and Bartunek (2005)	*eHealth and STS* Eason et al. (2012)	
		**Ergonomics work analyses**Considers the physical, cognitive, psychic and organisational aspects of work in an integrated way. This approach makes a distinction between task and activity							Wisner (1994)	*Service industry* Guerin et al. (2001)	
		**Variance analysis**Variance analysis aims at dealing with task interdependencies. A variance is an unwanted discrepancy between a desired state and an actual state or an unwanted or unexpected deviation from standard operating conditions or specifications		X			X		Rasmussen and Svedung (2000)	*Manufacturing job design*Davis and Wacker (1982)*Healthcare* Carayon, Alvarado, and Hundt (2003) and Montague and Kleiner (2009)	
STS theory, quality management, human factors and ergonomics, systems safety engineering	Macroergonomics *Variants*: STS Tavistock, action researchHuman systems integration (HSI)/manprint	**Macroergonomics methods***MEAD*This 10-step iterative process integrates STS theory and ergonomics to analysis and design the microergonomics and the work system's environmental and organisational characteristics and subsystems interfaces. Variances are evaluated to determine design constraints and opportunities for change	X	X	X		X		Davis and Wacker (1982)Manprint (1984), Kleiner and Booher (2003), Pew and Mover (2007), Hendrick and Kleiner (2001, 2002) and Kleiner (2003, 2008)	*Large complex technological systems; construction, healthCare, transportation* Kleiner (2006, 2008)	#1MedicalAdiminstration
		**Macroergonomic analysis of structure (MAS)** This method combines empirically developed analytical models of the effect of three major STS elements (technological subsystem, personnel subsystem and relevant external environment) on the fourth major element the structure of the organisation's work system. MAS analysis results can be compared with the existing structure of an organisation's work system to identify discrepancies for correction and design/redesign of the work system processes	X	X	X		X			*High technology organisations and university college*Hendrick and Kleiner (2001)	
		** SAT** A seven-step SAT that integrates STS and microergonomics to identify problems and probable causal factors as related to work systems. SAT is a process for developing strategic and systematic solutions to problems, developing potential solutions alternatives and evaluation of the costs/benefits trade-offs of potential solutions along with implementation strategies and effectiveness measures	X	X	X	X	X		Robertson (2002, 2005)	*Office ergonomics* Robertson (2002)*Workplace health and safety interventions*; Henning et al. (2009)	#4: Office ergonomics intervention
		**Organisational requirements definition for information technology (ORDIT)** ORDIT uses responsibility modelling as a basis for constructing sociotechnical systems opportunities. ORDIT constructs and evaluate sociotechnical scenarios				X			Eason, Harker, and Olphert (1997)	*Re-organisation of electricity company*Eason, Harker, and Olphert (1996)	
		**AcciMaps**AcciMaps are an accident analysis methodology that is used to represent graphically the causal factors involved in a particular accident or safety-compromising incident, occurring within complex sociotechnical systems. The approach also captures the preconditions and actions behind that causal chain of events. AcciMaps are diagrams developed to support vertical integration across the control levels of a sociotechnical system				X	X	Use of visual diagrams and models		*Healthcare* Waterson (2009a, 2009b); *Command and control*; Jenkins et al. (2010); *Outdoor accidents* Salmon et al. (2010); *Chemical accidents* Hopkins (2000)	
		**STAMP**STAMP views systems as consisting of interrelated components that are kept in a state of dynamic equilibrium by feedback loops of information and control. A system in this conceptualisation is not a static design – it is a dynamic process that is continually adapting to achieve its ends and to react to changes in itself and its environment		X		X		Modelling	Leveson (2012)	*Aviation and aerospace; rail transportation; public health,* Leveson (2012)	
	Simulations and modelling	**HI-TOP**HI-TOP (high integration of technology organisation and people) is a step-by-step manual procedure for understanding the impact of technological change on the organisational and human components. A simple tool that identifies the likely organisational and human implications of technology changes and implementation	X	X	X	X	X	Organisational readiness and technology assessment using critical technical features (CTFs)	Marjchrzak (1988a)	*Manufacturing*Majchrzak (1988b), Majchrzak et al. (2004)	
		**TOP modeller**This is a computerised decision support system for manufacturing organisations to assist decision-makers to identify the organisational changes required when new process technologies are being considered. Interdisciplinary teams models process variances, business objectives and gap analyses to prioritised the gaps to plan the technology and organisation redesign effort.			X	X	X	Gap analysis and priorities and rating of team agreement		*Manufacturing*Majchrzak (1997), Majchrzak and Gasser (2000)	
		**CIMOP system**A computer-integrated manufacturing, organisation and people knowledge base evaluation systems which focuses on evaluating computer-integrated manufacturing, organisation, and people system design				X	X	Two outputs: scale of results scales given numerical and verbal	Karwowski et al. (2002)	Karwowski et al. (2002)	
		**Microsaint**A general purpose, discrete-event simulation software tool with graphical user interface and flow chart. It is a flexible tool for optimisation where one can simulate what would happen if changes to the process occur				X			Laughery, Plott, and Scott-Nash (2007)	*Military, product design, health care, manufacturing, service industry*. Laughery, Plott, and Scott-Nash (2007)	
Experimental psychology	Multivariate conceptual scaling	**Psychological scaling***Multidimensional scaling (MDS)*MDS is a psychological scaling technique that begins with pairwise proximities for a set of items (concepts, names, steps, locations, etc.) and through multivariate analyses fits the proximities into N-dimensional space. The result is a spatial layout of items along dimensions thought to represent features which differentiate the items		X	X			Proximities	Kruskal (1977) and Kruskal and Wish (1978)	*Military*Schvaneveldt et al. (1985) and Gillan, Breedin, and Cooke (1992)	
		**Latent semantic analysis (LSA)** LSA is a computational linguistic model that represents language numerically, based on the generalised context in which words occur. Its main computational ramifications are that it can measure (a) proximities between text strings, and (b) amount of information contained in a text string. It is a useful way to compare large bodies of text (written or transcribed)						Collection of text	Landauer, Foltz, and Laham (1998)	*Military*Bolstad et al. (2007) and Freeman, Thompson, and Cohen (1999)	
		**Pathfinder**Like MDS, pathfinder begins with a set of pairwise proximities for items of interest and results in a graphical structure in which concepts are represented as nodes, and relations as links connecting the nodes		X	X			Proximities	Schvaneveldt (1990)	*Military* Cooke, Neville, and Rowe (1996), Cooke and Rowe (1997), Roske-Hofstrand and Paap (1986) and Schvaneveldt, Beringer, and Lamonica (2001)	
Experimental psychology	Cognitive, behavioural, organisational psychology	**Experiments and studies***Controlled experiments*Laboratory experiments include the testing of a research hypothesis that predicts causal effects of one or more variables; at least two levels of one or more independent variables, objective assignment of participants to conditions; systematic procedures for empirically testing hypothesised causal relationships and controls to reduce threats to internal validity		X	X	X		XHypotheses testing		Kerlinger (1986)	
		**Simulator studies**Experiments conducted using simulators or synthetic task environments		X		X	X	X(Models)		*Aviation, healthcare* Hollnagel (2012a, 2012b); *Nuclear powerplants* Burns et al. (2008) and Roth (1997)	
		**Field (quasi-experimentation)experiments**Method to measure the impact of an intervention on process and/or outcomes, generally conducted as a naturalistic or systematic observation without randomisation. Quasi-experimentation examines the deliberate and systematically manipulations of selected variables and the effect on the outcome or performance variables	X	X	X	X	X	Macro-ergonomics organisational questionnaire survey (MOQS)		Campbell and Stanley (1966)	#4: Office ergonomics intervention
STS theory, cognitive systems engineering and ergonomics	Participatory design and macroergonomics *Variants:* Cooperative design	**Design methodsPE**This approach to employee involvement that is concerned with ergonomics design and analysis. The involvement of people in planning and controlling a significant amount of their own work activities, with sufficient knowledge and power to influence both processes and outcomes to achieve desirable goals. With the implementation of technology in organisation, PE requires end users to be highly involved in developing and implementing the technology**Cognitive walkthrough**A *usability inspection method used to identify usability* issues focusing on how easy it is for new users to accomplish tasks with the system**Scenario-based design**Narrative descriptions of envisioned usage scenarios are used to enrich understanding of how practitioners might use the system being developed	X	X	X	X	X	Organisational and safety policies, procedure and practices	Noro and Imada (1991), Wilson and Haines (1997), Korunka, Weiss, and Karetta (1993), Carayon and Karsh (2000) and Carroll (1995)	*Oil industry; service industry; knowledge work*Imada (1991), Wilson and Haines (1997)*Software and computer systems*; Beyer and Holtzblatt (1998) and Carroll (1995)	
		**Affinity mapping**Representation and thematic technique. Used as a business tool to organise ideas, themes and dates. Adapted for use as part of contextual inquiry to organised notes and insights from field interviews	X	X		X		Visual diagrams of thematic related representations		*Computer systems; office environment design;* Beyer and Holtzblatt (1998) and Robertson (2002)	
Cognitive systems engineering		**Team interaction methods***EAST*This method combines hierarchical task analysis (HTA; Annett); coordination demand analysis; communications usage diagram; social network analysis propositional networks and an enhanced form of operation sequence diagram**Communication analysis**Examines the causes for remote requests (either from the point of view of an individual page, set of pages or individual source code line) and infers the access pattern						Network modelling	Stanton, Baber, and Harris (2008) and Walker et al. (2010)	*Air traffic controlMilitary*Stanton, Baber, and Harris (2008) and Walker et al. (2010)	#6: Sub-marine sound and control rooms
		**Interaction analysis**Methods for investigating interactions of human beings with each other and with objects or technologies in the environment; includes verbal communication, nonverbal interaction, the use of artifacts and technologies; identifying communication flow patterns and both static and dynamic patterns of interaction		X					Cooke and Gorman (2009)	*Military*Cooke et al. (2012) and Gorman et al. (2012)	
		**TARGETs**(targeted acceptable responses to generated events or tasks)Uses a behavioural checklist to record the occurrence or non-occurrence of responses to trigger events embedded in job-relevant scenarios		X		X				*Military teams*Fowlkes and Burke (2005)	
		**Team task analysis (TTA)** TTA typically consists of a set of scales which are used to quantitatively assess whether a job is individual or team-based. The method can also be used to assess aspects of team workflow and other patterns of work-based activities				X	X			*Military environments*Arthur et al. (2005)	
		**HTA-T (hierarchical task analysis for teams)** Uses a version of HTA specially adapted to analysing team tasks. This is combined with an event-related measurement scheme, which provides a set of objective criteria by which key team skills can be assessed		X			X			*Military teams (e.g. Navy)* Annett, Cunningham, and Mathias-Jones (2000)	
		**GTA (groupware task analysis)** Uses a combination of HTA and workflow (Swim lane) diagrams to understand the requirements and work context in which groupware systems can be introduced or used to support complex work tasks		X		X	X			*Security systems; information artefacts; large scale office systems*; van Welie and van der Veer (2003)	
		**TCTA (team cognitive task analysis)** TCTA is an adaptation of the Critical Decision Method (CDM; Klein and Armstrong 2005). The essence of the method is the conduct of a set of semi-structured interviews with all members of a team, using a predefined set of questions (probes) following observation of a task. A timeline of the task observed is developed noting the critical decision points. The interviews develop a view of the decision making requirements, cues used, reasons for difficulty, possible errors and strategies for effective decision-making	X	X			X			*Military applications*Klein (2000)	
		**Soft systems methodology (SSM)**SSM is a technique that was designed in order to facilitate the analysis of complex work systems (e.g. departments within a company, whole organisations involving many stakeholders) and to make it easier to carry out the task of planning and implementing change within these systems		X				Workshops and development of visual SSM diagrams		*Job design*Nadin et al. (2001) *Org. Dev.Manuf. and service* Checkland and Scholes (1999) and Clegg and Walsh (1998)	
Cognitive systems engineering; HFE	CWAVariants: applied CWA, work-centred design	**CWA**This is a functional analysis method that defines the goals, constraints and affordances in a domain that constitutes the cognitive problem-space that practitioners need to cope with – particularly to deal robustly under unanticipated conditions. CWA includes five interlinked analyses that focus successively on different layers of constraints ranging from characteristics of the work domain to individual strategies of domain practitioners to social and organisational factors through cognitive competencies	X	X	X	X	X	Document review	Rasmussen, Pejtersen, and Goodstein (1994)Vicente (1999)	*Healthcare*Burns, Enomoto, and Momtahan (2009), *Naval ship design* Burns, Bisantz, and Roth (2004)*Process control displays* Jamieson (2007) *Teamwork design* Naikar (2006)	
		**Applied CWA**A variant of CWA that uses a functional abstraction network as the basis for information requirements specification and representation design. Examples of CWA methods:	X	X	X	X	X		Elm et al. (2003)	*Command and control support*. Potter et al. (2003) *Software dev. and systems eng*; Elm et al. (2008)	
		**Work-centred design**Focuses on the demands and the broader context of the work							Eggleston (2003)	*Aviation planning* Evenson, Muller, and Roth (2008); Scott et al. (2005) and DePass et al. (2011)	
	Resilience engineering	**Functional resonance analysis method (FRAM)** FRAM provides a way to describe outcomes using the idea of resonance arising from the variability of everyday performance		X			X		Hollnagel (2012a, 2012b) and Hollnagel, Woods, and Leveson (2006)	*Healthcare*Perry and Wears (2012) *Financial services* Sundström and Hollnagel (2011)	
	Decision-centred designSituation awareness-oriented design	**CTAConcepts mapsCritical decision methodACTAGoal-directed task analysis**Interview and observation techniques designed to articulate cognitively challenging aspects of task; includes important contextual information such as information needs, goals and critical cues. CTA particularly focuses on capturing the knowledge and strategies that underlie expert performance, as well that distinguish experts from less-experienced performers. It also focuses on trying to understand the basis for errors	X X	XX		XX	XX	Analysis of critical incidents	Klein, Calderwood, and MacGregor (1989)Crandall, Klein, and Hoffman (2006)	*Damage control*: Miller et al. (2002) and Hutton, Miller, and Thordsen (2003); *Weather forecasting*: Hoffman Coffey, and Ford (2000);*Firefighting:* Klein, Calderwood, and MacGregor (1989)*Firefighting and electronic warfare*: Militello and Hutton (1998)*Biopharmacology* Kaber et al. (2006)	#3 UASs#5Colorectal cancer screening
		**Ethnographic methods/cognitive field studies**This method involves observations conducted in the actual task environment, coupled with semi-structured interview techniques that can occur in the actual task environment or in a more controlled setting	X	X				Focus groups	Nardi (1997)Roth and Patterson (2005)	*Power plants*Mumaw et al. 2000); *Railroads* Roth and Multer (2009); *Medical* Christian et al. (2006) and Patterson et al. (2006) *Crisis management* Militello et al. (2007)	#2Rail road operations#5 Colorectal cancer screening
		**Task analysis for error identification (TAFEI)** The method is used to evaluate design prototypes and provide a means by which errors can be predicted. The method makes use of HTA and a set of state space diagrams				X	X			*Consumer devices; human–computer interfaces* Stanton and Baber (2005) and Baber and Stanton (2001)	
		**Operator sequence diagrams (OSD)** OSD is a graphic representation of operator tasks as they relate sequentially to both equipment and other operators		X			X			Military; Aviation US Federal Aviation Authority (2012)	
		**Critical path analysis (CPA)** This project network technique uses a graphic network representation to depict the logical, temporal sequencing of activities		X			X			*Interface design.* Baber and Mellor (2001)	
Human factors and ergonomics, cognitive systems engineering, systems safety engineering	HRAHuman mental workload;	Probabilistic risk assessmentCognitive reliability and error analysis method (CREAM)Failure mode effects analysisSubjective workload assessment technique (SWAT)NASA task load index (NASA TLX)			X			Mapping	Kirwan (1994)Hollnagel (1998)Bowles and Bonnell (1993)Hart (1978)	*Military; oil industry; medical*;Railroad:Wreathall et al. (2004)Hollnagel (1998)Bowles and Bonnell (1993)Hart (1978)	
	Situation awareness	Team workload assessment; situation awareness global assessment technique (SAGAT)							Endsley (1988)	*Aviation, heathcare, oil industry:* Endsley (1988) and Endsley and Jones (2011)	

## 2. Research methods for STS and safety: an overview

### 2.1 Research traditions and methods

As is clear from Figure 1, many different approaches have been applied to the understanding of STS and safety. In order to focus on what we perceive to be some of the most influential over the years, we describe a set of main conceptual underpinnings which have guided traditions: STS theory, quality management, cognitive systems engineering, experimental psychology, human factors and safety engineering, and systems safety. It is possible to further sub-categorise these traditions into frameworks and approaches and specific methods. For example, cognitive systems engineering is associated with various types of approaches that include, among others, CWA and CTA. CWA can likewise be broken down into a number of sub-constituent methods (e.g. work domain analysis, control task analysis, strategies analysis, etc.). We recognise that our survey of STS methods for safety is partly hampered by the difficulty of identifying categories with which to allocate specific methods into discrete traditions and conceptual groups. There is a certain amount of ‘slipperiness’, for example, in distinguishing between methods and method ‘frameworks’. Many methods also fall into a number of traditions. As a result of this, we attempt to provide a representative, but not exhaustive, list of methods, as well as covering what we perceive to be the most important methods developed since the 1950s and 1960s. Table 1 describes the various traditions and their associated frameworks/approaches together with a (non-exhaustive) listing of representative methods (*n* = 52). Separate columns list some of the key references associated with each method alongside a brief description of each, the types of data collection techniques they involve, examples of their application, as well as cross-referencing the six case studies described in this paper.

### 2.2 Method selection

These frameworks and methods listed Table 1 were nominated for inclusion based on demonstrated real-world application via the experience of the authors, and/or methods published in the scientific literature (see Stanton et al. 2013; Carayon 2006; Karsh, Moro, and Smith 2001). Our intent was not to be exhaustive in describing all STS, Quality Management, HFE methodologies shown in Table 1. Primarily, our focus was on identifying those frameworks/methods that have emerged out of the discipline of STS and those that were inducted into the STS approach in the context of applied research. Many of the methods arising from traditions other than STS were selected because they effectively address a specific research context or research question relevant to system safety. Many of these are not as robust in describing interactions; however, they are appropriate for use in a specific context and defined system boundary. In contrast, the frameworks and approaches that are heavily invested in STS traditions and conceptual underpinnings provide methods designed to facilitate a better understanding of the context, boundaries and embedded interactions, such as the macroergonomics analysis and design of structure (MEAD) methodology (Kleiner 2003).

## 3. STS methods in action: six case study examples

Case studies represent an important means of illustrating the application of methods to real-world problems. It is often through the application of methods in complex settings that adaptation and innovation in method development occur. In this section, we discuss six case studies. These case studies showcase the range of methodologies that are employed in STS studies as well as the wide variety of domains to which STS methods have been applied. They also highlight that sociotechnical research often employs multiple methods to form a more complete picture, and that the methods employed are often adapted to the specific characteristics of the STS. We selected the case studies from a range of domains and with different project goals in order to illustrate the flexible application of methods in complex settings. An additional motivation for choosing these particular examples of ‘STS in action’ was to illustrate what each of the authors thought to be a particular strength of the STS approach within HFE, as well as highlight issues which are worthy of future considerations and/or extension (Section 5 of the paper). Each case study discussion in the following text emphasises the methods used and summarises aspects of the context in which the methods were used, types of data collection, analysis and representation of data, and outcomes from using the methods (Table 2).

**Table 2:** Order of case studies and summary of contents.

	Case study	Context	Data collection	Analysis	Representation	Outcome
1.	Medical administration	Comparison of two hospitals	Self-report study	Statistical contrast of factors (unit, step, situation and organisation)	Data tables and graphs	Units rather than hospitals had the greatest influence on safety violations
2.	Rail road operations	Coordination of trackside workers	Interview and observation	Assessment of customs and practices	Narrative descriptions	Informal ‘courtesies’ show how people adapt, and adapt to, the technology to their working practices
3.	UASs	Development of a synthetic task environment	Behavioural and cognitive interviews	CTA of workload and tasks	Coded interview responses	Understanding of crew coordination, training issues and human factors issues surrounding remote operation of UASs
4.	Office ergonomics intervention	Reducing work-related disorders and stress	Quasi-experimental longitudinal study: self-report surveys; interviews	Statistical contrast of groups (workstation, training and control); SAT; organisational training analyses	Data tables and graphs; coded interview responses; problem factors and objective activity trees; learning hierarchy; business decision scorecard	Demonstration that workstation and training conditions led to better performance and fewer symptoms/problems than the control condition
5.	Colorectal cancer screening	Tracking patient screening history and recommended actions	Ethnographic observation and CTA interviews	Coding of qualitative data to identify themes	Decision requirements tables and visualisations of new concepts	Display key patient information from multiple sources as an integrated visualisation
6.	Submarine sound and control rooms	Activities whilst returning to periscope depth	Audi recording and observation	Social network analysis of task, social and information networks	Task, social and information networks and combined networks	Understanding of the interrelations between people, tasks and information for team work and considerations for the next generation of systems

### 3.1 Case study 1: safety violations during medication administration

#### 3.1.1 Background and context

The first case study (Alper et al. 2012; Karsh, Waterson, and Holden 2014) illustrates the value of attempting to integrate macro- and microergonomics in order to assess the role of causal mechanism across a range of system levels (e.g. organisational-group-individual levels of analysis). For many years, there have been calls to better integrate microergonomic and macroergonomic research and practice (Scott and Charteris 2006; Zink 2000). Macroergonomics is about the design of entire work systems, and models have been proposed to help guide researchers in identifying salient job and organisational-level variables to study (Hendrick and Kleiner 2002; Smith and Carayon-Sainfort 1989). Few theories or models, however, explicitly provide causal pathways and mechanisms between organisational levels of the work system. The late Bentzi Karsh originally proposed the idea of *Mesoergonomics* as a way to specify macro- and microergonomic integration (Karsh 2006). Mesoergonomics has been defined as an open systems approach to ergonomics theory and research whereby the relationship between variables in at least two different levels or echelons is studied, where the dependent variables are human factors and ergonomics constructs.

Between 2004 and 2008, a set of studies was undertaken by Karsh et al. at the University of Wisconsin–Madison, the aim of which was to study the impact of a particular health information technology (barcoded medication administration) on nurse and patient outcomes (Karsh and Brown 2010). During that time, an opportunity arose to study safety violations during medication administration. Two urban, academic, tertiary-care, free-standing paediatric hospitals in the USA participated in the study. Hospital A had 222 beds and was located in the Midwest. Hospital B had 162 beds and was located in the South. Three units were studied in both hospitals: a paediatric intensive care unit (PICU), a haematology–oncology–transplant unit (HOT) and a general medical/surgical unit (Med/Surg).

#### 3.1.2 Methods used

In order to study safety violations, a self-report survey was designed and included the following items:

1. In actual practice, to what extent do you find yourself routinely having to break protocol for ‘*step*’? (Violation Situation: Routine)
2. In an emergency situation, to what extent do you find yourself having to break protocol for ‘*step*’? (Violation Situation: Emergency)

‘Step’ refers to the three steps of the medication administration process studied: matching the medication to the medication administration record (Match-MAR), checking a patient's identification before administering a medication (ChxID) and documenting the administration of a medication (DOC). Each nurse responded to all six combinations of questions (2 violation situations × 3 steps). Nurses responded using a seven-point scale (ranging from 0 – Not at all through to 6 – A great deal). There was also a ‘Don't Know’ option. A 4-factor mixed measures ANOVA was used to determine if the Hospital, Unit, Step and Situation impacted individual-level violations.

#### 3.1.3 Outcomes from using the methods

Statistical main effects were found for Unit, Step and Violation Situation, but not for Hospital. The null finding for Hospital is as important as the significant main effects; not finding hospital differences demonstrated that the findings were not isolated to what might otherwise have been labelled a problematic hospital. Instead, we found similar self-reported violation levels at two highly regarded paediatric hospitals. The data also showed that violations during emergency situations were more likely to occur than routine violations, that violations are more likely to be reported in the HOT unit, followed by the PICU, and then by Med/Surg units, and that violations were more likely to be reported in the ChxID process, followed by Match-MAR, and then by DOC. More importantly, there was a significant Unit ×  Process ×  Situation three-way interaction. The interaction revealed that there were no unit differences for Match-MAR or DOC, but for ChxID, the HOT units had significantly higher routine violation scores than the PICU and Med/Surg units. Also, in emergency situations, there were significant unit differences for all three steps of the medication administration process. For Match-MAR, the PICU and HOT units had significantly higher emergency violation scores than the Med/Surg units. For ChxID, all three units significantly differed from each other; the HOT units had higher violation scores than the PICU and Med/Surg units; the PICUs had significantly higher violation scores than the Med/Surg units. Finally, for DOC, the HOT units had significantly higher violations scores than both the PICU and Med/Surg units.

The results showed that although the hospital exerted no influence on individual violations, Units did. This is similar to other research in domains other than healthcare. Zohar and Luria (2005), for example, found in their study of safety climate in manufacturing that organisation-level and group-level climates were globally aligned; however, the data also revealed meaningful group-level variation in a single organisation, attributable to supervisory discretion in implementing formal procedures associated with competing demands like safety versus productivity. The hospital-based study also showed that the Unit with higher reported violations was the HOT unit, where patients often stay for weeks or months and the nurses get to know the patients. This led to the hypothesis that patient familiarity reduces nurses' perceived need to comply with medication safety protocols, perhaps thinking that they know the patients and their regimens sufficiently. The study provided the basis for a number of other hypotheses with patient familiarity as an independent variable. The results also demonstrated that another type of context variable – an emergency situation – increased the likelihood of individual level violations. One could further hypothesise that other emergency situations in other settings would have a similar effect because emergencies lead to time pressure and time pressure reduces the likelihood of complying with protocols that take extra time.

In summary, this case study illustrates the use of sociotechnical methods, in this case tailored surveys administered across units and organisations, to explore the role of causal mechanism across a range of system levels (e.g. organisational-group-individual levels of analysis). More particularly, it illustrates how sociotechnical methods can elucidate the multiple organisational and situational factors that can impact the likelihood of individual level violations that can have safety consequences.

### 3.2 Case study 2 – the role of informal practices in contributing to work system resilience

#### 3.2.1 Background and context

This case study, drawn from railroad operations (Roth, Multer, and Raslear 2006), reveals the importance of using sociotechnical methods, most particularly CTA, and field observation studies, to uncover and document features of the current environment that contribute to resilience as a foundation for developing next-generation technologies, so as to avoid inadvertently disrupting these strategies and degrading resilience.

Railroad operations require coordination among individuals widely distributed in space. This includes train crews; roadway workers who maintain the tracks, signals and related infrastructure; and dispatchers who manage track usage, allocating time on the track to different trains and roadway worker activity as required. These individuals rely heavily on analogue radio communication to maintain awareness of each other's location, coordinate work and maintain safe operations (Roth, Multer, and Raslear 2006). A goal of the research was to understand the factors that affect roadway worker safety in today's environment so as to anticipate the likely impacts of emerging technologies on roadway workers and to provide guidance for design and introduction of the technologies.

#### 3.2.2 Methods used

The study involved performing a CTA using a combination of structured interviews and field observations. Site visits and interviews were conducted at five locations in the USA and included passenger and freight rail operations. Included were sites where new technologies, targeted at roadway workers and train crews, were being piloted. Field observations included riding in locomotive cabs with train crews and accompanying roadway workers performing rail inspections. The structured interviews focused on understanding the cognitive and collaborative demands of the railroad work environment, particularly as they related to safety, and the strategies that train crews and roadway workers have developed for coping with task demands and contributing to safe operations. The interview protocol drew on standard CTA interview questions (e.g. applied cognitive task analysis (ACTA) – Militello and Hutton 1998) adapted for the railroad industry, and was designed to confirm and expand on the insights drawn from the field observations.

#### 3.2.3 Outcomes

Among the most notable findings of the study is that railroad workers have developed a variety of informal practices that, in combination, provide multiple layers of resilience contributing to safe operations. Roadway workers and train crews routinely exploit the ‘party-line’ aspect of radio communication to build and maintain awareness of the location, activities and intentions of others in their vicinity by ‘listening in’ on communication directed at others so as to anticipate and avoid potential collisions. For example, roadway workers actively monitor radio channels on which train crews communicate with each other in order to anticipate when trains are likely to approach them and in what direction so as not to be caught by surprise. This is particularly important in the case of unscheduled trains that may appear at a different time or from a different direction than expected.

Listening-in strategies also enabled railroad workers to recognise situations where information in their possession was relevant to the safety of others. The study also documented a number of instances where third parties played an instrumental role in preventing accidents. In several cases, individuals overheard conversations suggesting that someone was (erroneously) occupying track for which they did not have authority, or were about to be (erroneously) given authority to enter track that was already occupied. They immediately got on the radio to alert the parties of the potential conflicts. By intervening, they prevented a potential collision.

Observations and interviews also revealed proactive communication practices that went beyond the requirements of formal operating rules and served to foster shared situation awareness and enhance on-track safety. For example, dispatchers, train crews and other roadway workers routinely alert roadway workers of trains that may be about to reach them, particularly when these trains arrive at an unexpected time or from an unexpected direction. As one dispatcher stated, ‘I let them know what my plan is so that they are not startled’. Similar informal communications that provide an important safety function have been observed among train crews. For example, when a train crew passes a roadway worker group working by the side of the track, the conductor may call over the radio to alert other trains passing through the territory of the presence of the roadway workers.

Interestingly, these practices are referred to as ‘courtesies’ by the railroad workers, emphasising that they are not required by the operating rules, but rather are part of the informal redundant ‘safety net’ that is provided through voluntary proactive activities among railroad workers.

One of the dangers of introducing new technology is that it will disrupt effective strategies that contribute to system resilience. The case study highlights how insights gained from a field observation study can serve to guide new technology deployment so as to avoid these pitfalls.

Railroads are developing portable devices for roadway workers that integrate location-finding technologies (e.g. GPS) for more accurate location information and digital technologies for more reliable communication. They are also developing positive train control technologies that incorporate GPS location information and display-based digital communication devices. These new technologies have the potential to enhance mutual awareness of the location, activities, and intentions of trains and roadway worker groups within the distributed organisation, increasing overall safety. However, unless carefully deployed, they could disrupt the strategies that practitioners currently use to maintain shared situation awareness, resulting in a degradation of resilience. In particular, if digital technologies were deployed so as to eliminate the party-line aspect of current radio technology, without providing an alternative means to foster shared situation awareness, it could degrade the ability of individuals to maintain awareness of each other's locations, activities and intentions.

Properly deployed, location finding and digital communication technologies have the potential to reduce the challenges associated with analogue radio communications while still providing the kind of situation awareness information that is now extracted indirectly. For example, location-finding technology makes it possible to develop graphic displays that directly show the location of roadway workers and trains in a given vicinity. The same information display could be made available to dispatchers (on a display in the dispatch centre), roadway workers (on portable graphic devices) and train crews (on a display in the locomotive cab). Thus, location information that is important for shared situation awareness, which is now extracted indirectly (e.g. by listening in to radio communications directed at others), could be obtained more directly and with lower cognitive overhead.

In summary, this case study illustrates how sociotechnical methods, most particularly CTA and field observation studies, can be used to uncover and document features of the current STS that contribute to safety and resilience. It also points to the use of such methods to inform the design of next-generation technologies, so as to avoid inadvertently disrupting effective strategies and degrading resilience.

### 3.3 Case study 3: the case of unmanned aerial systems

#### 3.3.1 Background and context

Unmanned aerial systems (UASs) are not without controversy. Interestingly, there has been a fair amount of controversy simply surrounding the label. Today, the US Air Force (USAF) calls them Remotely Piloted Aircraft, yet most of the military recognises the UAS label. For the purpose of this discussion, it is relevant that the term Unmanned Aerial Vehicle is now considered passé by some in favour of UAS. The movement from ‘vehicle’ to ‘system’ recognises the fact that the UAS is so much more than an air frame, although one may not realise that in the UAS exhibit halls. The UAS cockpit is basically on the ground, and called the Ground Control Station or Operator Control Station. In addition, there are numbers of individuals on the ground doing UAS-relevant tasks of launch and recovery, maintenance, navigation, sensor operation, imagery analysis, mission planning and the list goes on. The UAS is definitely a system and, arguably, a system of systems. Current plans to have swarms of autonomous vehicles and to have single vehicles integrated into the National Airspace further expand the scope of this system and make salient the need for safety.

UASs have primarily been used by the military and this is a domain where it is notoriously difficult to gain access for observations, interviews or other data collection. USAF scientists carried out an early attempt to understand the users' tasks in the UAS environment. They conducted a CTA for the purpose of developing simulation environments (Gugerty et al. 1999).

#### 3.3.2 Methods used

Seven Predator AVOs (Air Vehicle Operators or pilots) as well as 12 operators familiar with sensor and mission planning jobs were interviewed. The team also conducted behavioural interviews asking specifically about each operator's task and workload. The recorded audio from the interviews was transcribed and coded and represented in terms of a goal hierarchy for Predator missions. From this information, the USAF scientists designed a synthetic task called the BRUTE Recon Task which they used to understand more about Predator performance parameters. This same analysis has been used to construct other synthetic environments (CERTT, CERTT-II, Cooke and Shope 2004).

Other data collection efforts have taken place incrementally and rather informally, through interviews with subject matter experts. For instance, our team organised several annual Human Factors of UAVs workshops to which we invited operators. We held Q and A sessions in which significant information was elicited (Pedersen et al. 2006). Some more formal structured interviews have been conducted with Army sensor operators for the purpose of understanding the expertise associated with imagery analysis relevant to identification of improvised explosive devices. Interviews and responses to written surveys were coded and analysed to identify the most frequently mentioned static and dynamic cues and this information was used to build training materials for novice sensor operators (Branaghan, Cooke, and Staszewski 2012; Cooke et al. 2010).

#### 3.3.3 Outcomes

Altogether, these data have been useful for creating synthetic task environments, for developing training materials, and for guiding laboratory research on issues relevant to the task. Some of the earliest human factor issues identified from the various data collection efforts included questions of appropriate training for operators, problems of spatial disorientation of pilots, fatigue because the vehicles are often high endurance, restricted so-called ‘soda-straw’ views of the world from a camera and Ground Control Station human interface issues.

One could conclude that the CTA led to some successful products and was critical in identifying important issues for operator safety. This would be the case. But interestingly, a new set of issues has appeared that are coming from functional needs of the user (the government or big user, not the operator). The new issues are of a different flavour and include concepts of multivehicle control by a single operator, swarming of multiple autonomous vehicles, and integration of UAS into the National Airspace. These issues are at a level beyond the early CTAs; they are at the macrocognitive or system of systems level and indeed cross multiple levels.

In addition to these recent demands from the user, we have observed some interesting cases of what one might call local success, but global failure. This can be the case when one focuses on the design of a part of a system, only to neglect the system at other levels and the interactions with those levels. For instance, one of the authors has heard an anecdote about fielding a new laptop controller in the Middle East. The laptop worked well in its control of the UAS by a single operator; however, when the operator in the test saw something of interest, there was no way of communicating that to the soldiers in harm's way. Indeed, it is often the case that the focus is on the control of the UAS, only to forget that the main task is one of reconnaissance. Similarly, with the increasing evidence on autonomous swarms and multi-vehicle control, the capacity to collect data dramatically increases. There is little attention to the apparent system bottleneck which is the sensor operator and imagery analysts, at the pointy end of the stick, pouring over screens of video data 24/7. Thus, the automation serves to save manpower in one part of the system only to greatly overload the humans at another. These new issues cross traditional system boundaries and require a look much broader than the traditional focus on one or two operators at a workstation. The earliest work was not intended to address these types of issues and it remains to be seen whether the same CTA methods can adequately address them.

In summary, this case study illustrates the value of sociotechnical methods, such as CTA, in identifying important issues for operator safety and informing the design of realistic simulations and a test-bed for further analysis and design. At the same time, it points to the challenges confronting the STS community as the focus of analysis shifts from individuals and teams to larger organisations and systems of systems.

### 3.4 Case study 4: effects of a macroergonomics longitudinal intervention on knowledge workers' health and performance

#### 3.4.1 Background and context

Teamwork that fosters positive group dynamics to conduct organisational activities and meet business goals is essential to organisational effectiveness in complex, computer-based work environments. However, the importance of effective teamwork is underscored by the occurrence of work-related musculoskeletal disorders (WMSDs) and psychological stress among knowledge workers (e.g. Carayon and Smith 2000). Concerns exist about the escalation of these computer-related WMSDs, given the high prevalence of WMSDs among computer users (between 40% and 80%) and the growing global computer workforce (e.g. Tittiranonda et al. 1999; Katz et al. 2000). To mitigate these adverse health trends and enhance work effectiveness, a STS approach is desirable in order to integrate the macro- and microergonomics components and design effective interventions for computer-based workers (Robertson and Courtney 2004; Haims and Carayon 1998).

This case study describes how macroergonomics served as our organisational design approach to reduce the health and safety risks and increase performance by providing flexible physical work environments and accommodating the ergonomics needs of individual employees and project teams. Emphasis is placed on participation of workers in the workspace design process and training in the optimal use of the workspace as a tool for safe and effective work (Haims and Carayon 1998). Enhancing individual workers' control over their work environment allows them to influence decisions about where and how they might work (McLaney and Hurrell 1988; Robertson and Huang 2006), leading to improved physical health and performance (Karasek and Theorell 1990). Ergonomics training is another fundamental element of our macroergonomics approach, as it can integrate ergonomics into an organisation. The value of incorporating a macroergonomics approach to design an office ergonomics organisational intervention and the longitudinal effects it has on employees' health and performance are illustrated in this case study.

#### 3.4.2 Methods used

We employed a macroergonomics approach to design an office intervention which included four important work systems elements: workspace flexibility, training, user involvement and management commitment. In designing the intervention, we used the results of the systems analysis tool (SAT) (see Tables 1 and 2) reported in Robertson and Courtney (2004). These results provide a systematic view to better understand the underlying causal factors of the health and performance issues associated with office and computer work, and how to effectively solve them (Robertson and Courtney 2004). We designed a longitudinal field study to investigate the effects of this intervention on musculoskeletal health, the psychosocial work environment, and work effectiveness in a computer-based office setting.

We used a quasi-experimental, non-randomised, study design in which one pre-intervention survey (two months prior) and two post-intervention surveys (three and six months after) were used to collect data from the experimental and control groups (Campbell and Stanley 1966). The interventions consisted of: (1) a new workspace with adjustable workstations and a flexible overall facility layout that included a variety of conveniently located meeting spaces of different sizes and (2) office ergonomics training that encouraged employees to exert control over how the workspace was used. We expected that ergonomics training, coupled with flexible workspace design, would maximise effectiveness for the workers. Moreover, these intervention effects might translate into behavioural changes (for example, re-arranging/adjusting workspaces, changing computing work habits), thereby leading to a reduction in musculoskeletal discomfort and an increase in psychosocial work environment factors, environmental satisfaction, group performance and business process efficiency. The participants were assigned to one of the following groups: (1) flexible workspace-only (WS-only, *n* = 121), (2) flexible workspace + training (WS+T, *n* = 31) and (3) no-intervention control (*n* = 45). Management defined four goals for the workplace intervention project: (1) create a new concept for work environments that enables greater worker effectiveness, (2) provide ergonomically designed workspaces that enhance employees' health and well-being, and support employees' and teams' job tasks by being adaptable to the changing work process through flexible, moveable and adjustable workspaces, (3) increase communication and collaboration among individuals, groups and departments and (4) create operational efficiencies through business process effectiveness. Facility and ergonomics teams were established that led several employee Workplace Change Communication initiatives and conducted workspace ergonomics needs analyses. An instructional design model based on a systems approach was used to create the office ergonomics training which consisted of five processes: needs analysis, design, development, implementation and evaluation (Kirkpatrick 1979). It was critical in the design phase to conduct a thorough needs assessment at the organisational, group and individual levels to determine appropriate design of the training and aligning the goals with the organisational strategies (Robertson 2002). To measure the impact of the intervention, four methods of data collection were employed: (1) Work Environment and Health electronic surveys, (2) Ergonomics knowledge tests, (3) Observations and (4) business process analysis (BPA).

#### 3.4.3 Outcomes

This longitudinal, field office ergonomics intervention study found overall, positive, significant effects on outcome variables for the two intervention groups compared to the control group. Eight of the 10 outcome variables (workspace, lighting, privacy, job control, collaboration, corporate culture, ergonomic climate and communication) were significant, where the Workstation-only and Workstation + Training groups improved over time as compared to the control group. In two of the psychosocial outcome variables, the Workstation + Training group showed significant improvements by reporting a higher sense of community (communicating corporate culture) and ergonomic climate than the Workstation-only and control groups over time. Improvements in the WMSDs were revealed for both the Workstation-only and Workstation + Training groups compared to a control group, with the Workstation + Training group experiencing a greater reduction in WMSDs for seven body parts. Over 60% of the intervention groups responded that they agreed or strongly agreed with being involved in the design process. The business processes analyses (time and costs) demonstrated positive effects on quantitative measures of organisational output for both the Workstation-only and Workstation + Training groups.

The Workstation + Training group participants were encouraged through the training to use corporate resources to achieve an ergonomic fit with their new workstations. This group's ergonomics awareness appears to be further supported by the observed high level of reported agreement in understanding ergonomics principles and that management listens to and acts upon employees' needs related to office ergonomics. This trained group also reported a higher sense of belonging to the organisation and a more positive sense of community with the organisation than the Workstation-only group, indicating that the driving force may not have been solely the workplace redesign, but these important values being conveyed by management as delivered in the training. The finding that the Workstation + Training group exhibited significant decreases in WMSDs compared to the control and Workstation-only group suggests that training acts synergistically with the workspace changes and provides employees with the knowledge necessary to change and adjust their workstations to fit their postures and workflow needs, and thus reduce the risk of musculoskeletal discomfort. The observation that the Workstation-only group reported a greater decrease in overall body discomfort relative to the control group suggests that providing ergonomic furniture alone may be beneficial; however, without coupling training with the new workstations, strong overall health benefits were not observed.

In summary, this study provided compelling evidence that the macroergonomics intervention was effective among knowledge workers in office settings and should be considered in future preventive workplace initiatives (Robertson et al. 2008). It illustrates the use of quasi-experimental methods and multiple dependent measures to assess the impact of interventions at multiple levels, including musculoskeletal health, psychosocial satisfaction and work effectiveness.

### 3.5 Case study 5: visualisations to support colorectal cancer screening

#### 3.5.1 Background and context

This case study highlights a project combining CTA, ethnographic observations and simulation-based studies to develop tools to support primary care providers based in the USA in tracking colorectal cancer screening for their patients. Colorectal cancer screening is difficult to track because screening happens at different intervals depending on the type of testing used and the risk factors present for an individual patient. For example, for a person with no additional risk factors, colonoscopy is recommended every 10 years, or flexible sigmoidoscopy is recommended every 5 years or faecal occult blood testing is recommended every year. If risk factors are present, or the patient has had abnormal findings in the past, recommended intervals change. Thus, the primary care provider must keep track of which test the patient has had in the past (if any), what findings were present (if any) and relevant risk factors in order to make recommendations about when the next testing should occur. This should be relatively simple with electronic health records and the use of computerised clinical reminders. The problem is exacerbated, however, by the fact that much of the testing occurs outside of the primary care clinic, so previous findings may or may not be available in the electronic health record. Furthermore, the primary care provider may need to search several locations within the record (progress notes, consult notes, pathology reports, gastroenterology reports, etc.) to find the information needed to make a recommendation about when the next colorectal cancer screening test is due.

#### 3.5.2 Methods used

A series of studies began with a project to identify barriers and facilitators to colorectal cancer screening (Saleem et al. 2009). This initial study included two days of ethnographic observation at each of four sites in which investigators observed primary care providers discussing colorectal cancer screening with their patients, as well as relevant intake and discharge procedures. Opportunistic interviews were conducted when possible with primary care providers. Findings from this study guided the design of a notional visualisation that would integrate all the information relevant to a patient's colorectal cancer screening into one screen. A simulation study was conducted to evaluate the potential value of the visualisation concept (Saleem et al. 2011).

A second study used CTA interviews to deepen on the information needs and decision strategies used by experienced primary care providers in both public and private health systems. Findings from this study guided more detailed design of a visualisation to support colorectal cancer screening and surveillance (Lopez and Militello 2012).

#### 3.5.3 Outcomes

The findings from these studies suggest that primary care providers need three different categories of information at different times. The first category includes two key pieces of information related to colorectal cancer screening needed for every patient: (1) they need to know whether the patient is in screening or surveillance mode and (2) they need to know when the next recommended test is due. In many cases, particularly those in which the patient has no additional risk factors and is adherent to the recommended screening regimen, this information is sufficient.

A second category includes screening history. In some cases, the primary care provider needs to be able to quickly review the patient's screening history. This may give the provider a sense of the patient's adherence in the past, which may in turn influence the tone of the conversation. A historical view may also provide important information about prior findings. Even non-cancerous growths can change the recommended screening intervals.

A third category includes broader contextual information and reference materials. Information such as the patient's risk factors, conditions that may impact the patient's ability to tolerate a colonoscopy, medications that may interfere with a colonoscopy and screening guidelines are needed only occasionally.

These findings point to a relatively simple visualisation to support colorectal cancer screening and surveillance decision-making. It is feasible to design an application that will query the electronic health record to extract the needed data to populate the visualisation without additional data entry. Although not all the relevant data may be available in the electronic health record, having confidence that the available data is visible will save time and frustration associated with searching. Furthermore, with the advent of health information exchanges, populating the visualisation with data from a range of sources (i.e. pathology lab, gastroenterology clinic) is an increasingly tractable problem.

We anticipate that the application of CTA and other STS approaches will continue to be critical as electronic health records and clinical decision support technologies become increasingly pervasive. Widespread introduction of relatively new information technologies across the healthcare system offers great potential for increased efficiencies and decision support technologies to increase patient safety. However, to realise these benefits and avoid unintended negative consequences, it will be important to explore the sociotechnical impacts during design phases, as well as during integration of new technologies and beyond.

In summary, this case study highlights the use of multiple sociotechnical research methods, including CTA, ethnographic observations and simulation-based studies to identify barriers in the current environment that get in the way of adoption of safety-relevant practices, in this case colorectal cancer screening. It illustrates how these sociotechnical methods can be used to inform design of new decision-support technologies to overcome those barriers.

### 3.6 Case study 6: event analysis of systemic teamwork

#### 3.6.1 Background and context

This project was undertaken as an ‘information audit’ of the relationship between the sound room and control room on board a submarine. The purpose was to understand how information flowed around the system in order to determine what requirements might be included in the next generation of tactical systems. The initial analysis focused on the task of returning the submarine to periscope depth. The decision to return to periscope depth (RTPD) is continually being assessed and reassessed. If an unexpected contact appears in the area where the submarine is heading, or is heading towards that area, then the decision to RTPD will be cancelled and the submarine will head down to the safe depth (below that of the deepest ship's hull, i.e. greater than 30 metres). The focus of the sound room and control room is on identifying all of the contacts surrounding the area where the submarine is intending to RTPD. This focus continues all the way up and when the ‘look’ (using the periscope) is established. At any point in the manoeuvre, the ship will return to a safe depth if a contact is detected. The decision to RTPD begins with the officer of the watch (OOW) calling an outstations briefing to ensure all people and systems on the submarine are in order and ready for the manoeuvre. This is followed by ballasting (to ensure the submarine is in trim) and clearing of stern arcs (so that the sound room can check for vessels behind the submarine). Then the sound room and control room are engaged in the activities to range all contacts within the local area to find a safe area of sea to RTPD. If no safe area is found, then the submarine will need to change area and continue to range contacts. When all contacts have been ranged and an area of sea has been identified, the OOW reports to the Captain for permission to RTPD. If permission is forthcoming, the OOW will request final reports from outstations to check that the people and systems on the submarine are in order and ready for the manoeuvre. A decision will be made by the OOW whether to conduct the standard routine (where range and bearing of all contacts are called out as the submarine returns to periscope depth) or silent routine (where the submarine returns to periscope depth as stealthily as possible). At periscope depth, the OOW calls ‘breaking’ and conducts two sweeps with the periscope to check that the submarine is clear from potential collision with contacts, and then will range the contacts manually to update the submarine maritime command system (SMCS) operators. Then the mission intentions can be carried out. If the submarine is on a potential collision course with any of the contacts, the OOW will give the order to dive to the safe depth.

#### 3.6.2 Methods used

**Figure 2:**
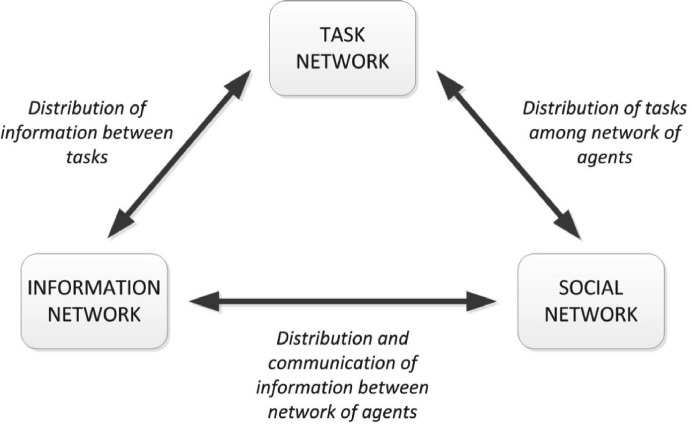
The complex collaborative systems of EAST and its underlying networks approach.

Event analysis of systemic teamwork (EAST; Stanton, Baber, and Harris 2008; Stanton 2014) offers a way of characterising command and control in multilayered networks. Command and control scenarios are characterised by multiple individuals and teams working together in pursuit of a common goal (comprising multiple interacting sub-goals). EAST provides a framework of methods that allow collaborative performance to be comprehensively described and evaluated (Stanton, Baber, and Harris 2008). Since its conception, the framework has been applied in many domains, including land and naval warfare (Stanton et al. 2006), aviation (Stewart et al. 2008), air traffic control (Walker et al. 2010), railway maintenance (Walker et al. 2006) and the emergency services (Houghton et al. 2006). EAST is underpinned by the notion that complex collaborative systems can be meaningfully understood through a network of networks approach (see Figure 2). Specifically, three networks are considered: task, social and information networks. Task networks describe the goals and subsequent tasks being performed within the system. Social networks analyse the organisation of the system (i.e. communications structure) and the communications taking place between the actors working in the team. Finally, information networks describe the information that the different actors use and communicate during task performance (i.e. distributed situation awareness).

The EAST framework lends itself to in-depth evaluations of complex system performance, examination of specific constructs within complex STS (e.g. situation awareness, decision-making and teamwork), and also system, training, procedure and technology design. While not providing direct recommendations, the analyses produced are often highly useful in identifying specific issues limiting performance or generating system redesign recommendations.

A major advantage of networks is that they do not differentiate between different types of node (e.g. artefacts and/or people) so that from a modelling perspective, they are not constrained by existing structures of people and artefacts, and rather they are related to the tasks associated with a scenario. It is possible to model the temporal aspects of networks by identifying critical moments in the sequence of activity. To do this, the scenario is divided into task phases allowing active and non-active elements to be specified and represented.

The EAST showed how the task, social and information networks can model the activities between the sound and control rooms. The nine outputs, namely the task network, the association matrix, the social network (and associated metrics), the communications classifications, the information network, and the combined networks (i.e. task and social networks, information and social networks, and task, social and information networks) offer a graphical representation of the activities in the sound and control rooms from different perspectives. The different perspectives offered by the representations are an attempt to characterise the activities between the sound and control rooms in returning the submarine to periscope depth. The representations offer different views with varying levels of fidelity. As with the analyses from CWA, the benefit of the EAST models is to help understand what is going on between the sound and control rooms, and how people share information related to the tasks. The ultimate goal of the work is to model alternatives and provide metrics for choosing one alternative over another.

#### 3.6.3 Outcomes

EAST described the sound room and control room activities in terms of task, social and information networks as well as exploring the relationships between those networks. The individual networks were used to describe the respective relationships between the tasks (such as the task dependencies and sequences), between social agents (such as sociometric status of agents based on communications), and information (such as the interdependences between the concepts discussed). An analysis of the communications revealed how different roles were transmitting different categories of information. The combined task and social networks showed which roles were performing the tasks in series and parallel. The combined information and social networks showed which roles were communicating the information concepts. The three integrated networks described how information was used and communicated by people working together in the pursuit of tasks. Any new concepts of the command system will need to consider the likely changes on these sociotechnical networked structures.

In summary, the EAST method was able to characterise the activities in the control room of the Trafalgar class submarine. The case study illustrates how sociotechnical methods such as EAST can be productively applied to a complex sociotechnical ‘system of systems’. The EAST networks offer multiple perspectives on the activities in a system which is a necessary requirement for sociotechnical analysis. The EAST method can be used not only to characterise existing systems but also to explore and compare alternatives as ideas for design of the social and technical aspects of the system co-evolve.

EAST does this by changing the task, social and information networks and their interdependencies. Adopting this sociotechnical systems design approach would help to jointly optimise the whole system rather than the parts in isolation. This would require spending more time in the initial modelling and prototyping, working with end-users and SMEs with more focus on the social systems and ways of working (to redress the balance of focus on technical system development) than is currently the case.

## 4. An evaluation of current methods for STS and safety

Section 3 showcased a sampling of case studies to illustrate the wide range of successful methods and approaches that fall within the broad STS umbrella. The case studies illustrate the breadth of safety applications to which STS methods have been applied, ranging from individual practitioners to multi-level organisations. At the same time, they suggest increased challenges as the scope and boundary of the system under study expand (e.g. to include ‘systems of systems’). In this section, we describe an evaluation study we conducted to further explore the strengths and weaknesses of current STS methods with the aim towards identifying gaps and future needs.

### 4.1 A framework for evaluation

The methods described in Table 1 were categorised into seven groups (macroergonomic methods, simulation and modelling methods, methods which made use of psychological scaling, experimental studies, design methods, team interaction methods, and cognitive task/work analysis). Each of the ‘families’ or groups of methods were evaluated against a set of seven broad criteria:

1. The extent to which the method examined aspects of work tasks
2. The extent to which the method examined aspects of the work domain
3. The extent to which the method represented individual, team and organisational concerns
4. The extent to which the method examined aspects of the wider environment/context
5. The types of outcomes produced by the method
6. The robustness of the method (e.g. validity, reliability)
7. The usability and support requirements of the method

Each of the seven categories contained a set of sub-questions relating to theoretical and practical aspects of the coverage of a method as it related to STS and safety.

### 4.2 Evaluation procedure

The authors of this paper, along with other experts drawn from either participants at the Hopkinton conference or contacted by email by the authors (total = 15), completed the categories and questions which were presented in the form of an Excel spreadsheet. Participants were asked to rate the extent to which they were familiar with the method families. For those method families with which they were familiar, participants provided ratings of 1–5 (5 = YES it is perfect for this, 4 = MOSTLY, could be used in this way, 3 = TO SOME EXTENT, a stretch but could work; 2 = SELDOM, would be unlikely; cannot think of any examples; and 1 = NO, never) in response to each of 33 queries. These queries were also categorised into seven generic dimensions of methods.

### 4.3 Results

**Figure 3:**
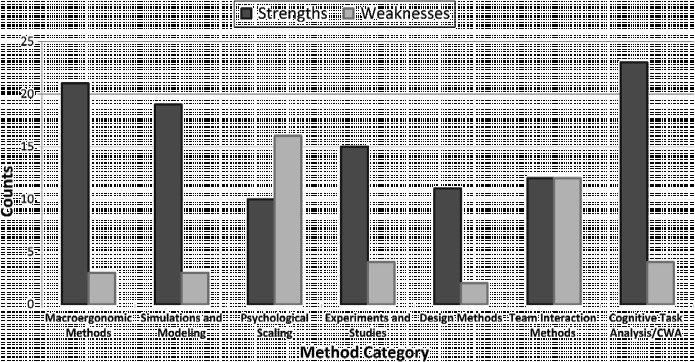
The number of strengths and weaknesses across the 33 dimensions for each ‘family’ of methods.

The number of respondents who were familiar with the method families is shown in Table 3. At least five of the respondents were familiar with each method. The median rating was calculated for each of the 33 dimensions across the respondents. Median ratings of 2 or less were interpreted as weaknesses of the method ‘family’, whereas median ratings of 4 or greater were interpreted as strengths. Figure 3 indicates the number of strengths and weaknesses across the 33 dimensions for each ‘family’ of methods. Macroergonomic methods, simulation and modelling and CTAs were viewed by respondents as having the most strengths, whereas psychological scaling and team interaction methods were viewed as the weakest. It should be noted that the latter two methods are also of more special purpose than the three stronger sets of methods.

**Table 3:** Participant ratings of familiarity with methods.

Method ‘family’	Method	Number of participants
Macroergonomic methods	Action research	8
	Ergonomic work analyses	
	Variance analysis	
	MEAD	
	MAS	
	PE	
	SAT	
Simulations and modelling	Functional resonance analysis	12
	ORDIT	
	AcciMaps	
	STAMP	
	HI-TOP	
	TOP MODLER	
	Microsaint	
Psychological scaling	MDS	5
	LSA	
	Pathfinder	
Experiments and studies	Controlled experiments	15
	Simulator studies	
	Cognitive field studies	
	Field (quasi-experimentation) experiments	
Design methods	PE	11
	Cognitive walkthrough	
	(SSM)	
	Affinity mapping	
Team interaction methods	EAST	7
	Communication analysis	
	Interaction analysis	
CTA/CWA	Abstraction–decomposition matrix	9
	Decision ladders	
	Goal-directed task analysis	
	Concept maps	
	Resilience engineering	
	Critical decision method	
	ACTA	
	Applied CWA	
	Cognitive field studies	
	Social organisation and cooperation analysis	
	Strategies analysis	
	Control task analysis	
	Worker competencies analysis	

**Figure 4:**
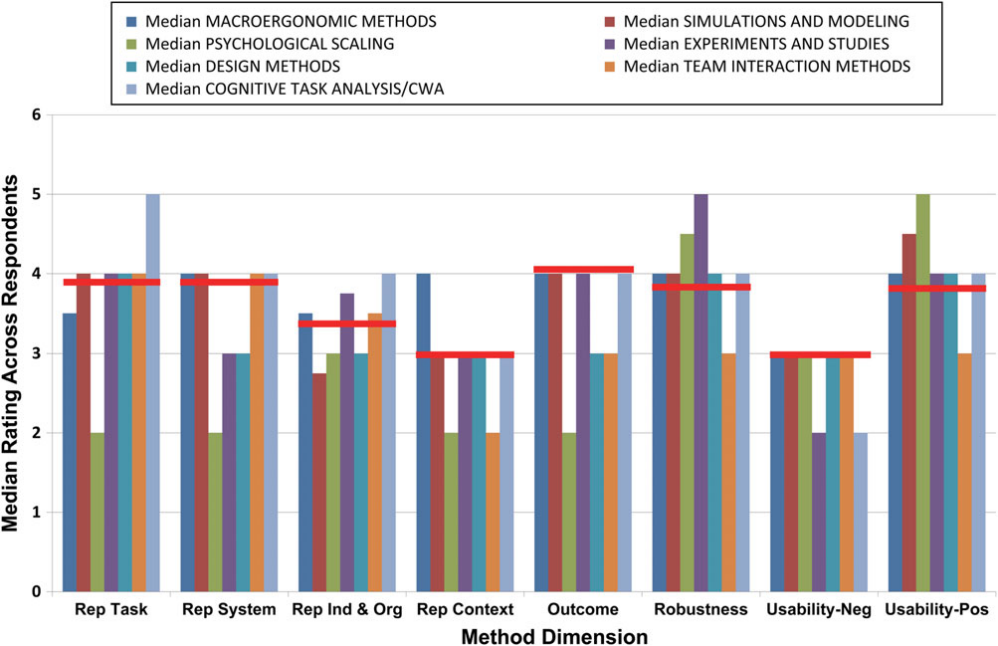
The medians for each method category and dimension.

In order to identify general gaps across all methods, the ratings were further collapsed across queries by taking the median rating for the queries within each dimension. Medians for each method category and dimension are displayed in Figure 4. As a whole, the methods tended to be weakest at representing context and on usability in terms of the resources required (expertise, analytic time) to apply the methods.

## 5. Discussion

In this section, we first reflect on our survey of the range and types of STS methods which currently exist before moving on to describe some of the outstanding challenges and unresolved issues which might form the basis of future theoretical and methodological work. We base our reflections, challenges and suggestions for this work on Table 1, our case studies (Section 3), the results of the expert evaluation (Section 4), as well as our own judgments of some of the issues which are likely to become prominent in the next decade or so. A summary of some of the most important directions for future research is given in Table 4.

**Table 4:** Summary of future directions for research and practice.

	Implications	
Issue	Theory	Practice
Defining what is meant by a STS approach to safety	Identifying the core constructs and elements of STS, and applying this to safety are often difficult	STS means many things to different people and this can sometimes be confusing, especially for safety practitioners
	It is difficult to pin down how the various theoretical traditions which make up STS relate to one another	‘Navigating’ through the vast range of methods is often difficult for non-experts
	Many of the most established constructs within STS (e.g. optimisation, redundancy) need to be reappraised in the light of more recent theory	
The coverage of STS and its application to safety	Current methods do not address external and environmental aspect of the work domain (e.g. regulatory, economic influences on safety)	A narrow range of causal factors may be used to explain the nature of accidents and human error
	The boundaries between system elements are often blurred or insufficiently defined	
The usability of current methods	There is not enough support in current methods to analyse interactions across system levels	Interventions which are designed on the basis on using a method may be unsuccessful, since safety may involve a number of system levels and the interaction between these
Methods reliability and validity		Many methods prove to be difficult to use, time consuming and require a lot of training
		Few methods describe details covering their reliability and validity

### 5.1 Current STS methods: some reflections

The 52 methods described in Table 1 were generated by the authors on the basis of either having used the method ourselves, or of reading about its use by other researchers. Table 1 is not meant to be exhaustive and no doubt we could have added many more methods to our survey of current practice. The breadth of the methods described in Table 1, alongside the number of studies carried out using them and the wide variety of application domains, is in many ways an indication of the success of the STS and its application to safety. In part, this reflects the fact that STS has, over the years, attracted the attention of researchers and practitioners from a wide range of disciplines (e.g. organisational psychology, industrial relations, human factors/ergonomics, and human resource management). The methods also draw on the full range of qualitative and quantitative approaches spanning ethnography through to controlled, field- and laboratory-based experiments. Most of the methods make use of multiple sources of data collections (e.g. surveys, interviews, task analysis). In some cases, four or five different data collection methods are often used in parallel (e.g. participatory ergonomics (PE), CWA). Again, this reflects some of the main strengths of the STS approach, namely the desire to provide a ‘holistic’ assessment of work–system interfaces and to capture the interaction between these (e.g. human–job, human–machine – Hendrick and Kleiner 2001, 2002; Waterson 2009a, 2009b; Singleton 1971).

In addition to reflecting some of the strengths of the STS approach to safety, Table 1 also demonstrates some of the problems associated with drawing boundaries in terms of what qualifies as an STS method and what falls outside of this body of tradition. The term ‘sociotechnical systems’ has been adopted by a variety of different disciplines (e.g. human factors/ergonomics, systems engineering, computer science) and is reflected in the history and origins of the methods. Many of these disciplines use the term in a precise manner, as seen in the development of STS principles for systems design (e.g. Clegg 2000). In other cases, the use of the term by other disciplines is much looser and hard to pin down (e.g. within systems engineering – Leveson 2012). In many ways, this may not be a problem (i.e. STS supports interdisciplinarity within the broad remit of ‘safety science’). In other cases, it can prove to be confusing, not only in terms of the goal of achieving theoretical coherence across disciplines, but also for practitioners who may want to know something about the theoretical assumptions built into a specific STS method relative to others. In the remainder of the paper, we explore some of these theoretical and practical issues in more depth.

### 5.2 STS methods: some theoretical challenges

One of the outcomes from the expert evaluation (Section 4) was that most STS current methods prove to be weak at representing the environment and context of the work system. Wider environment issues such as the role of political, legislative or regulatory factors in shaping the overall functioning and mode of operation of work systems do not appear to be addressed by most methods. To some extent, this is not surprising as it is a gap which has been discussed by other authors (e.g. Wilson et al. 2009; Rasmussen 2000). We note that CWA addresses these issues; however, the tools within CWA require further development and much more work could be done examining the role played between ‘external’ system influences (e.g. political and economic influences) and organisation, group and individual levels of analysis. This is possibly an area in which we can learn from other disciplines (e.g. policy studies, organisation studies) as well as an area in which there is scope for future development. In terms of representing the context, another outcome from the expert evaluation was that some methods and frameworks such as CWA, CTA and macroergonomic (e.g. MEAD) are strong at representing the task, the work domain, and the individuals and organisation. This may be, however, not surprising since these methods are really frameworks for analysis that include multiple specific methods to cover different types of analyses. This contrasts markedly with methods such as ‘multi-dimensional scaling’ that have a narrower ‘niche’.

Most current methods also struggle with the issue of delineating and distinguishing between system boundaries (cf., case study 1). This is an area which has also been mentioned in the past as a future direction for research. Over 20 years ago, Wilson and Grey (1990) posed the following questions in their summary of research on work design: Where do we draw the boundaries of what constitutes a work redesign initiative (e.g. task, job, role, environment and organisation levels)? and What are the key factors at each level and how do they interact? These questions are still important and we have a long way to go before we are in a position to answer them fully. Current methods provide only minimal support in identifying some of these key issues and the range of influencing factors which may exist across levels of analysis. Many methods focus on specific levels (e.g. group or team factors), but do not facilitate consideration of the influence of cross-level factors. This represents a gap in our current understanding of the holistic properties of STS (Wilson 2014).

Finally, while many of the methods clearly derive from theoretical traditions (Figure 1, Table 1), it is not clear how these relate to one another. One possibility is that future work in this area could focus on trying to seek commonalities across STS methods. Part of this is likely to involve examining some of the earliest work in STS (e.g. the Tavistock tradition – Weisbord 2012; Trist and Bamforth 1951), as well as more recent theories which seek to explain the social, organisational and technological issues involved in complex work domains (e.g. actor/network theory – Latour 1992, 1999; distributed cognition – Hutchins 1995; structuration theory – DeSanctis and Poole 1994). Much of the theoretical work within STS was developed in the 1950s and 1960s and many of the core constructs and components (e.g. optimisation of social and technical systems, the concepts of system redundancy) need to be reappraised in light of these new theories and concepts.

### 5.3 STS methods: implications for practice

An important emphasis from the earliest days of work within STS is the importance of using methods to provide systematic and informed judgments regarding improvements to workplace safety and employee well-being (Weisbord 2012). Part of this involved actively encouraging and involving workers in participative decision-making regarding changes or improvements to their work environments (e.g. the use of action research). Throughout the history of STS, there has, likewise, been a strong tradition which emphasises the importance of the practical application of methods and their use by non-experts or practitioners. One of the outcomes from our evaluation of current methods (Section 4) was the fact that many methods are viewed as difficult to use, time consuming and requiring a lot of training.

Table 1 also demonstrates that there are a vast amount of STS methods in the public domain. Indeed, as was pointed out earlier, the methods listed in Table 1 represent a small subset of available methods (see Pew and Mover 2007; Stanton et al. 2004 for more comprehensive compendiums of methods). This might be seen as a positive feature of work in the STS tradition – i.e. researchers and practitioners have a great deal of choice about the types of methods they can use as well as choosing a specific method to suit a particular context. On the other hand, it may prove to be a source of confusion for those less familiar with previous research within human factors/ergonomics and safety science more generally. In short, what we have is a lot of tools, methods, framework, etc., but not much help in trying to piece together the parts of the ‘jigsaw’. One direction for future work would be developing guidance to help practitioners ‘navigate’ through the various traditions and theories underpinning different types of methods. Such an endeavour would be useful for researchers as well. In preparing our survey of current methods (Table 1), we found that it was not always straightforward to decide on the ‘roots’ of a specific method. Likewise, and perhaps more concerning, it is not always possible to specify how a method works (i.e. what procedural steps are involved).

We also note that there appears to be some duplication of effort with some families of methods (e.g. macroergonomics). Some methods appear on the face of it to carry out similar analyses and possibly produce very similar results. Further comparisons of the use of two or more methods together, using a common scenario or work-related problem would be useful in pinning down the exact nature of differences, limitations and the advantages of one method over another. Many methods (e.g. CWA) are made up of different components which can be used to analyse separate aspects of work systems and safety issues. This type of modularity has a number of advantages, particularly in terms of saving analyst time and effort. Further work on how different methods could be combined, or broken down into parts that could be used in tandem with other methods, might also be a fruitful direction for research.

Finally, in a number of cases, we were struck by the lack of studies of the reliability and validity of methods. Do STS methods provide an accurate picture of workplace safety? How do separate results obtained from two (or more) analysts compare? Although there is a tradition of carrying out work in this area (see for example Burns, Bisantz, and Roth 2004; Hoffman et al. 2002; Gordon, Flin, and Mearns 2005; Boring et al. 2010; Waterson, Older Gray, and Clegg 2002), much more work is needed, particularly as it relates to the reliability of qualitative outputs of many methods.

### 5.4 STS methods and safety: some final thoughts

During the process of carrying out our survey of current STS methods for safety, we were impressed by the range and scope of the different types of methods which have been developed over the course of the last 50 or so years. Considerable work has been carried out within this area and, as we have suggested above, this has advantages and disadvantages in terms of the coherence of STS as a whole. What is, perhaps, needed more than anything else are attempts to consolidate this body of work, particularly as it relates to the development of methods. STS offers valuable insights into the nature of safety and accident causation; however, there is also a pressing need to attempt to unify, or at least seek commonalities from the point of view of theory and practice, across the research traditions described in this paper.
